# Supplementary material for: BMP-9 Modulates the Hepatic Responses to LPS
Source: Cells. 2020 Mar 4;9(3):617. doi: 10.3390/cells9030617 (PMC7140468; doi:10.3390/cells9030617)
Supplement: Supplementary file 1 [file cells-09-00617-s001.zip › Suppl_Fig4_Rev2.pptx]

## Slide 1
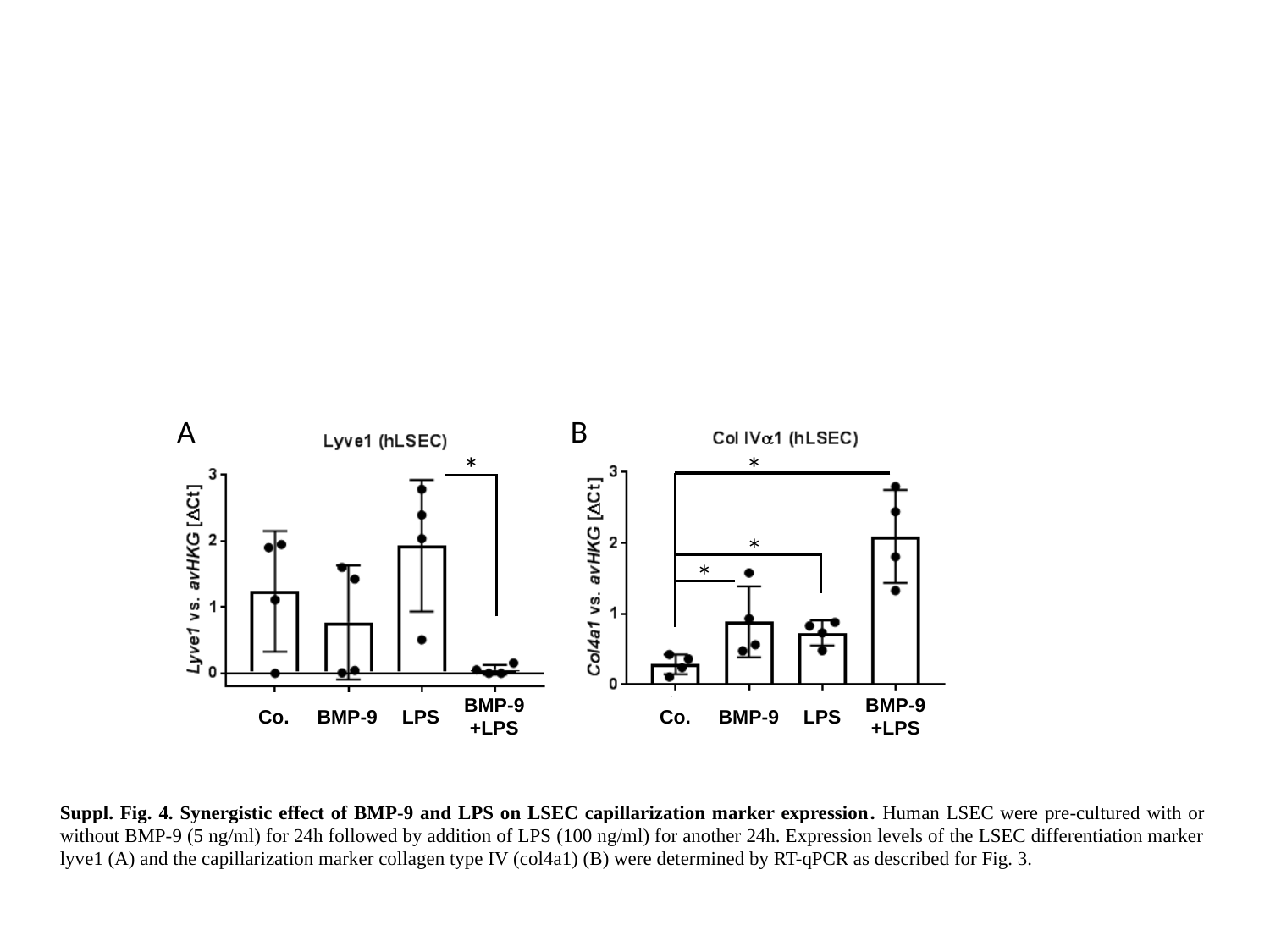

A
B
*
*
*
*
BMP-9 +LPS
BMP-9 +LPS
Co.
BMP-9
LPS
Co.
BMP-9
LPS
Suppl. Fig. 4. Synergistic effect of BMP-9 and LPS on LSEC capillarization marker expression. Human LSEC were pre-cultured with or without BMP-9 (5 ng/ml) for 24h followed by addition of LPS (100 ng/ml) for another 24h. Expression levels of the LSEC differentiation marker lyve1 (A) and the capillarization marker collagen type IV (col4a1) (B) were determined by RT-qPCR as described for Fig. 3.
