# Supplementary material for: BMP-9 Modulates the Hepatic Responses to LPS
Source: Cells. 2020 Mar 4;9(3):617. doi: 10.3390/cells9030617 (PMC7140468; doi:10.3390/cells9030617)
Supplement: Supplementary file 1 [file cells-09-00617-s001.zip › Suppl_Fig_1_Rev2.pptx]

## Slide 1
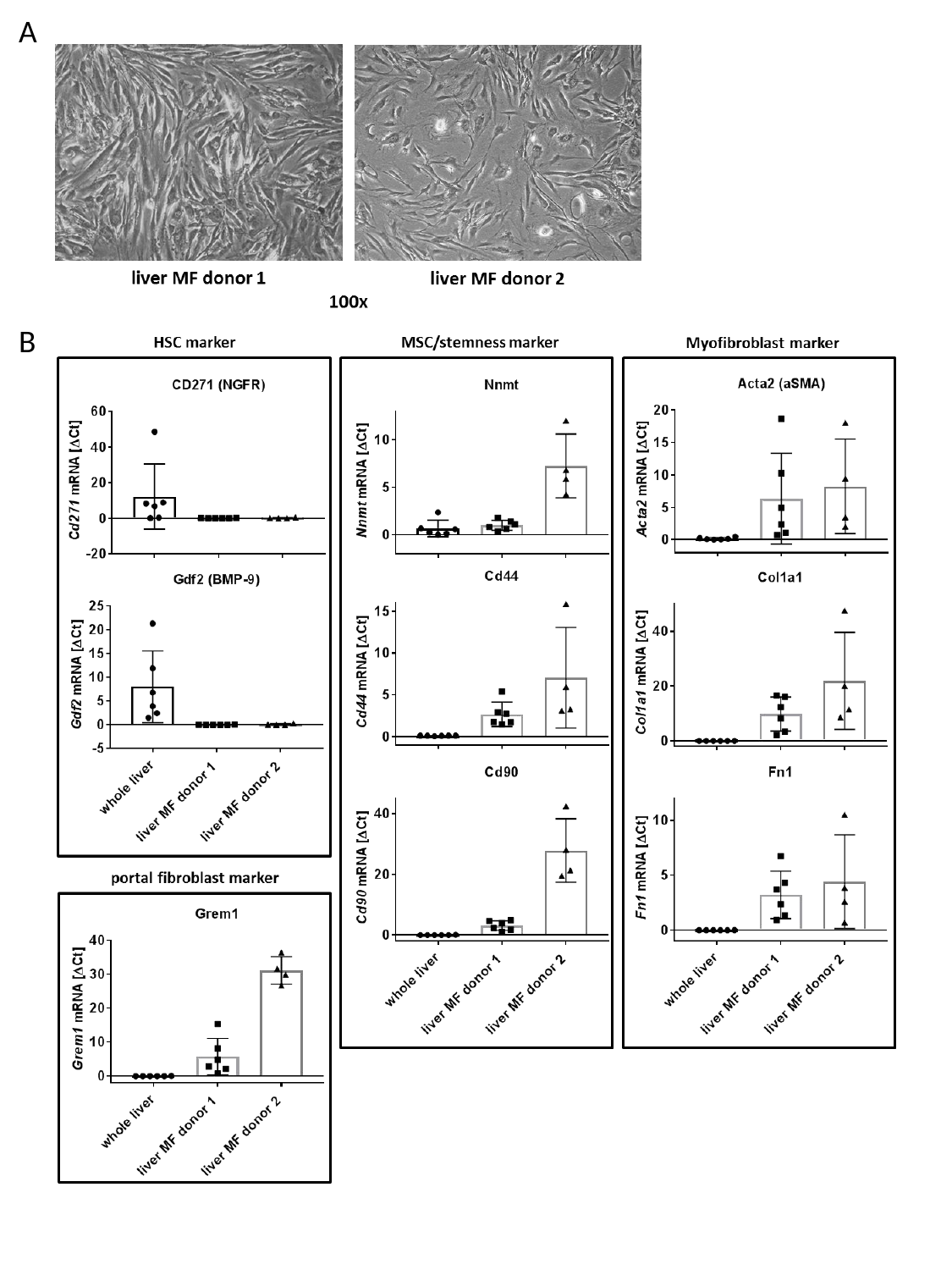

A
B

## Slide 2
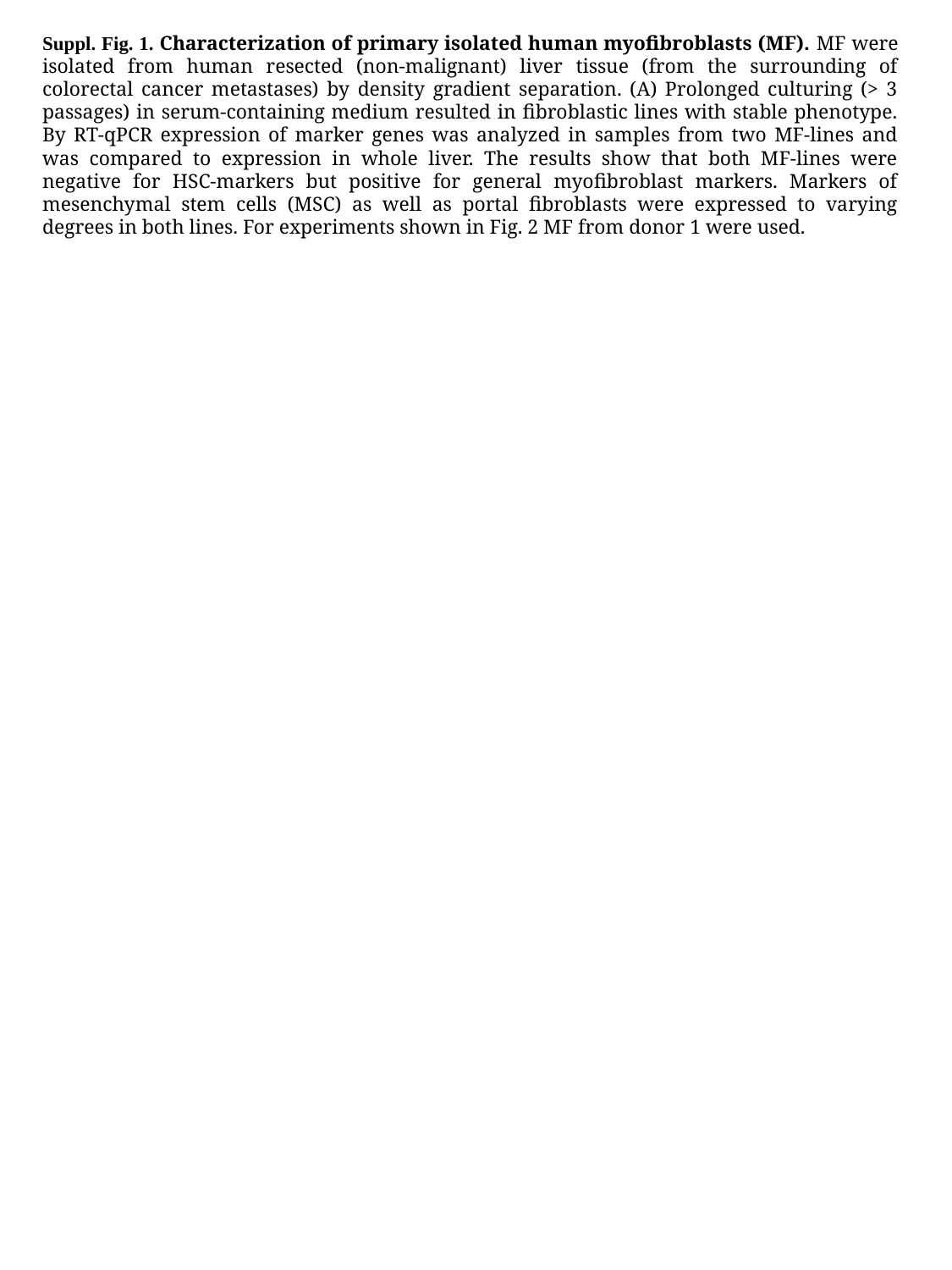

Suppl. Fig. 1. Characterization of primary isolated human myofibroblasts (MF). MF were isolated from human resected (non-malignant) liver tissue (from the surrounding of colorectal cancer metastases) by density gradient separation. (A) Prolonged culturing (> 3 passages) in serum-containing medium resulted in fibroblastic lines with stable phenotype. By RT-qPCR expression of marker genes was analyzed in samples from two MF-lines and was compared to expression in whole liver. The results show that both MF-lines were negative for HSC-markers but positive for general myofibroblast markers. Markers of mesenchymal stem cells (MSC) as well as portal fibroblasts were expressed to varying degrees in both lines. For experiments shown in Fig. 2 MF from donor 1 were used.
