# Supplementary material for: BMP-9 Modulates the Hepatic Responses to LPS
Source: Cells. 2020 Mar 4;9(3):617. doi: 10.3390/cells9030617 (PMC7140468; doi:10.3390/cells9030617)
Supplement: Supplementary file 1 [file cells-09-00617-s001.zip › Suppl_Fig2_Rev2.pptx]

## Slide 1
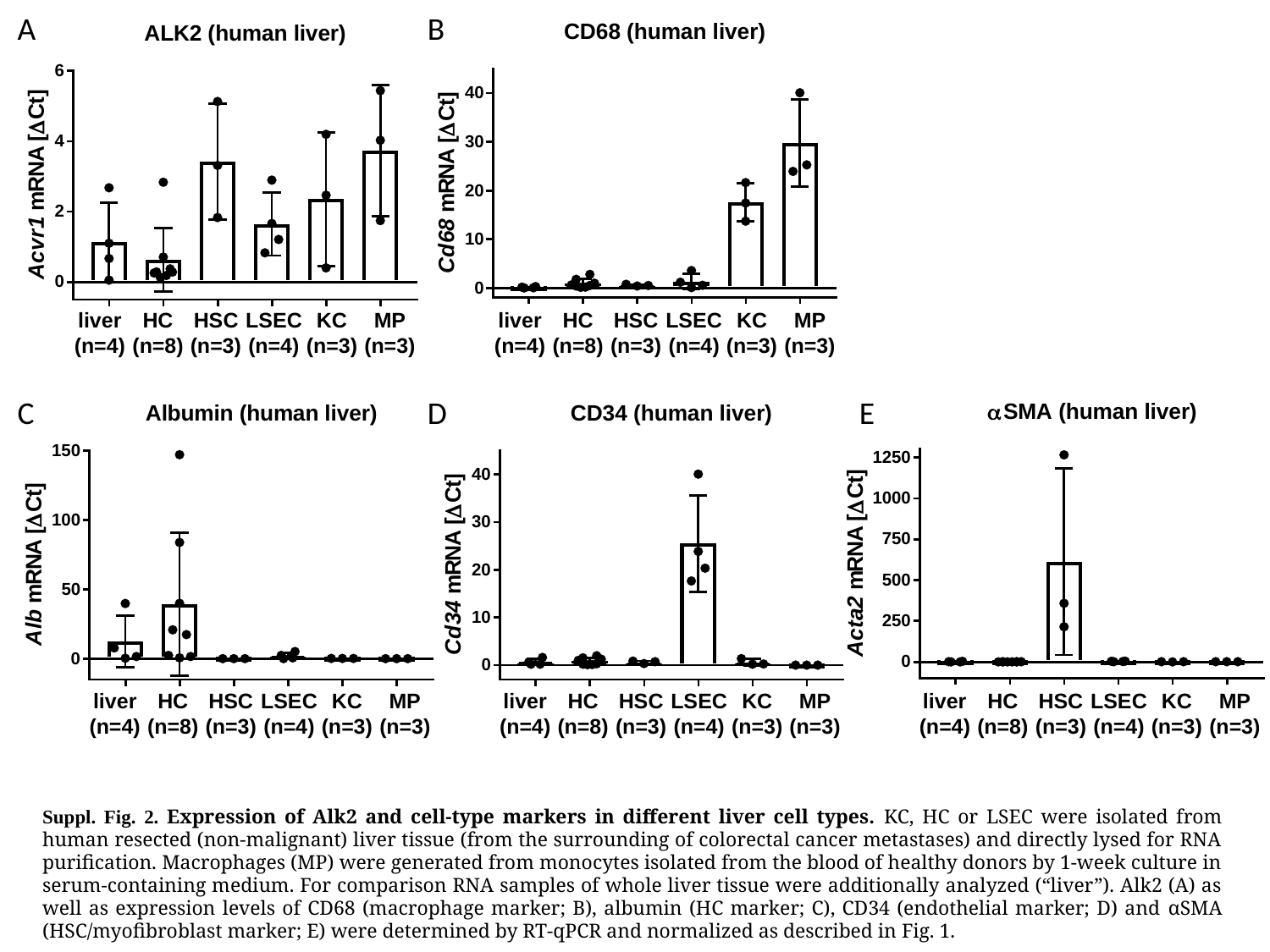

A
B
liver
(n=4)
HC
(n=8)
HSC
(n=3)
LSEC
(n=4)
KC
(n=3)
MP
(n=3)
liver
(n=4)
HC
(n=8)
HSC
(n=3)
LSEC
(n=4)
KC
(n=3)
MP
(n=3)
C
D
E
liver
(n=4)
HC
(n=8)
HSC
(n=3)
LSEC
(n=4)
KC
(n=3)
MP
(n=3)
liver
(n=4)
HC
(n=8)
HSC
(n=3)
LSEC
(n=4)
KC
(n=3)
MP
(n=3)
liver
(n=4)
HC
(n=8)
HSC
(n=3)
LSEC
(n=4)
KC
(n=3)
MP
(n=3)
Suppl. Fig. 2. Expression of Alk2 and cell-type markers in different liver cell types. KC, HC or LSEC were isolated from human resected (non-malignant) liver tissue (from the surrounding of colorectal cancer metastases) and directly lysed for RNA purification. Macrophages (MP) were generated from monocytes isolated from the blood of healthy donors by 1-week culture in serum-containing medium. For comparison RNA samples of whole liver tissue were additionally analyzed (“liver”). Alk2 (A) as well as expression levels of CD68 (macrophage marker; B), albumin (HC marker; C), CD34 (endothelial marker; D) and αSMA (HSC/myofibroblast marker; E) were determined by RT-qPCR and normalized as described in Fig. 1.
