# Supplementary material for: BMP-9 Modulates the Hepatic Responses to LPS
Source: Cells. 2020 Mar 4;9(3):617. doi: 10.3390/cells9030617 (PMC7140468; doi:10.3390/cells9030617)
Supplement: Supplementary file 1 [file cells-09-00617-s001.zip › Suppl_Fig3_Rev2.pptx]

## Slide 1
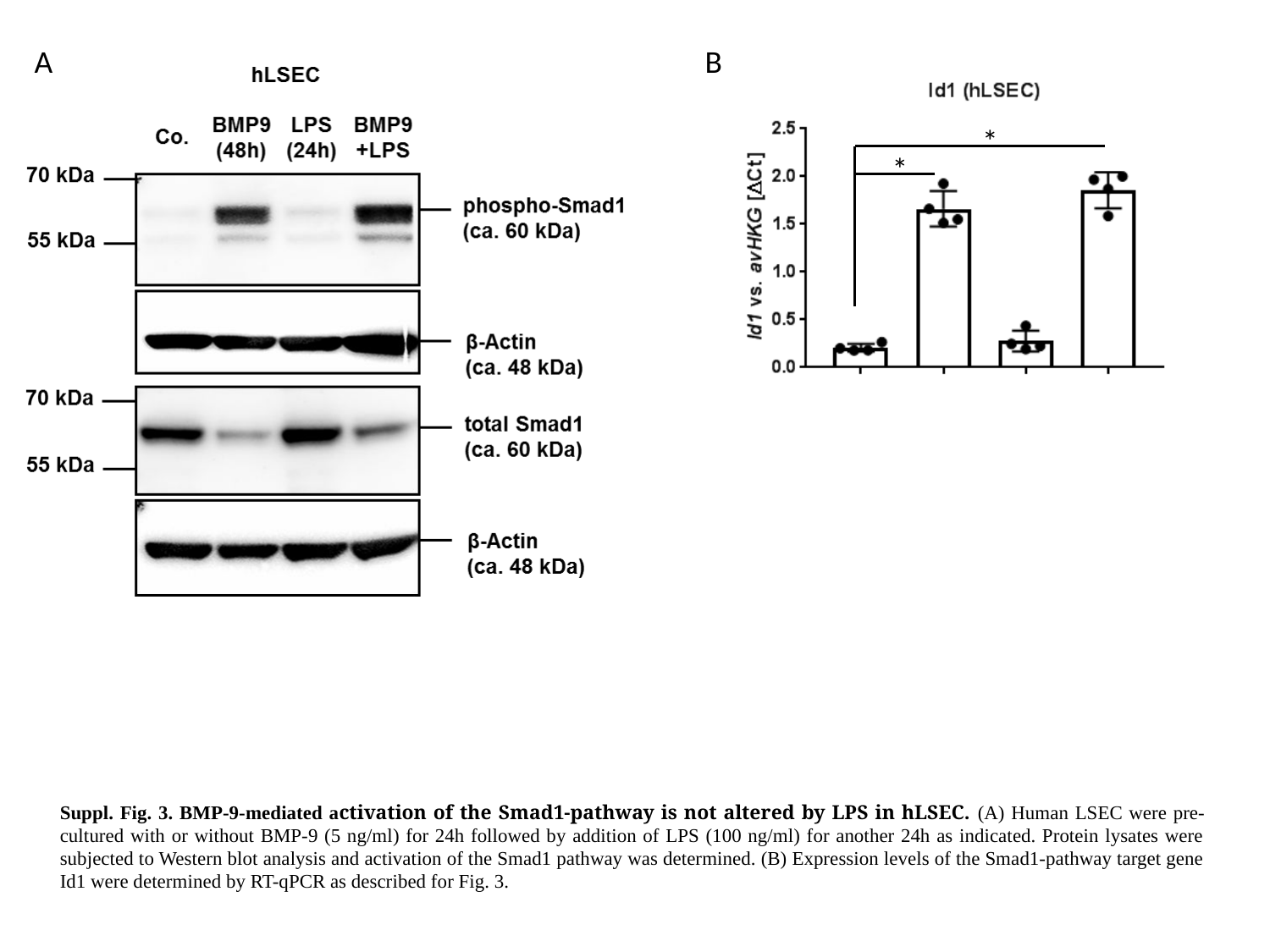

A
B
*
*
Suppl. Fig. 3. BMP-9-mediated activation of the Smad1-pathway is not altered by LPS in hLSEC. (A) Human LSEC were pre-cultured with or without BMP-9 (5 ng/ml) for 24h followed by addition of LPS (100 ng/ml) for another 24h as indicated. Protein lysates were subjected to Western blot analysis and activation of the Smad1 pathway was determined. (B) Expression levels of the Smad1-pathway target gene Id1 were determined by RT-qPCR as described for Fig. 3.
